# Investigation of the change in myocardial blood flow by perfusion CMR after revascularisation of chronically occluded coronary arteries

**DOI:** 10.1186/1532-429X-13-S1-P168

**Published:** 2011-02-02

**Authors:** Arshad Zaman, Nigel J Artis, A Crean, David L Buckley, Steven Sourbron, Sven Plein, John P Greenwood

**Affiliations:** 1 Medical Physics, Leeds, UK; 2 Academic Unit of Cardiovascular Medicine, Leeds, UK

## Introduction

The perceived clinical benefits of undertaking revascularisation to coronary artery chronic total occlusions (CTO) relate to improvements in angina symptoms and prognosis^1,2^. Long term survival benefits from CTO revascularisation has been suggested in several large observational studies despite a significant failure rate^1,3^. Data about the physiological consequences of successful opening of a CTO are limited and heterogeneous^4^. CMR imaging can provide quantitative information on MBF (myocardial blood flow) in this context.

## Purpose

To determine changes in hyperaemic myocardial blood flow in patients undergoing revascularisation of CTO.

## Methods

Twenty patients with CTO were recruited from clinical waiting lists and underwent perfusion-CMR before and after attempted CTO revascularisation on a Philips 1.5 T Intera system. Perfusion imaging was performed every heartbeat during the first pass using a T1-weighted fast (spoiled) GE sequence in 3 short-axis imaging planes. Stress perfusion imaging was performed using IV adenosine for 4 minutes (at 140mcg/kg/min) and 0.05 mmol/kg of gadolinium chelate via a power injector. The dynamic contrast enhanced image data were post-processed off-line using the software PMI 0.4^5^. Following motion correction, a circular ROI was selected in the left ventricle to measure the arterial input function. MBF maps were created by model-free analysis. Two myocardial ROIs were drawn on these maps, one in the CTO territory and one in a remote normal region. MBF for these ROIs was calculated using the Fermi model^6^. Statistical calculations were performed using SPSS.

## Results

**Figure 1:**
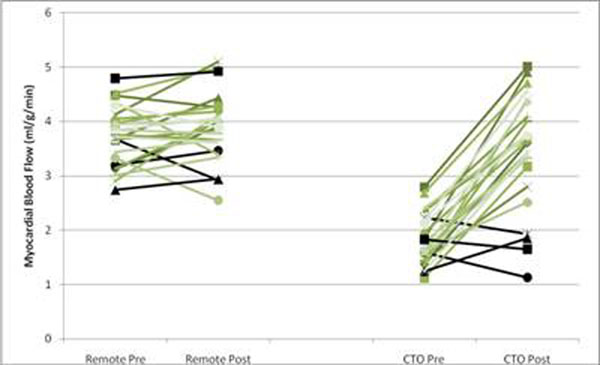
Graph of myocardial blood flow in the remote (normal) regions versus reperfused regions (the Black lines represent the 4 subjects that were not revascularised).

In 16 of the 20 patients, revascularisation was successful but failed in 4. There were highly significant improvements in adenosine-stress myocardial perfusion in the perfusion territories of the revascularised vessels. The improvements in myocardial blood flow were confined to the segments that were revascularised (p<0.001), whilst there were no changes seen in the remote myocardium (p=0.176). Figure 1 demonstrates the improvement in myocardial blood flow in the CTO versus remote regions.

## Conclusion

Stress perfusion CMR demonstrates significant improvements in hyperaemic MBF in patients with successful revascularisation of CTO.
